# Progesterone receptor membrane component 1 is phosphorylated upon progestin treatment in breast cancer cells

**DOI:** 10.18632/oncotarget.19819

**Published:** 2017-08-02

**Authors:** Marina Willibald, Giuliano Bayer, Vanessa Stahlhut, Gereon Poschmann, Kai Stühler, Berthold Gierke, Michael Pawlak, Harald Seeger, Alfred O. Mueck, Dieter Niederacher, Tanja Fehm, Hans Neubauer

**Affiliations:** ^1^ Department of Obstetrics and Gynecology, University Hospital and Medical Faculty of the Heinrich-Heine University Duesseldorf, Duesseldorf, Germany; ^2^ Molecular Proteomics Laboratory, BMFZ, Heinrich Heine University Duesseldorf, Duesseldorf, Germany; ^4^ Institute for Molecular Medicine, University Hospital Duesseldorf, Duesseldorf, Germany; ^3^ NMI Natural and Medical Sciences Institute at the University of Tuebingen, Reutlingen, Germany; ^4^ Department of Women's Health, University Hospital and Faculty of Medicine of the Eberhard Karls University Tuebingen, Tuebingen, Germany

**Keywords:** breast cancer, PGRMC1, progestins, norethisterone, casein Kinase 2

## Abstract

Menopausal hormone therapy, using estrogen and synthetic progestins, is associated with an increased risk of developing breast cancer. The effect of progestins on breast cells is complex and not yet fully understood. In previous *in vitro* and *in vivo* studies, we found different progestins to increase the proliferation of Progesterone Receptor Membrane Component-1 (PGRMC1)-overexpressing MCF7 cells (MCF7/PGRMC1), suggesting a possible role of PGRMC1 in transducing membrane-initiated progestin signals.

Understanding the activation mechanism of PGRMC1 by progestins will provide deeper insights into the mode of action of progestins on breast cells and the often-reported phenomenon of elevated breast cancer rates upon progestin-based hormone therapy. In the present study, we aimed to further investigate the effect of progestins on receptor activation in MCF7 and T47D breast cancer cell lines. We report that treatment of both breast cancer cell lines with the progestin norethisterone (NET) induces phosphorylation of PGRMC1 at the Casein Kinase 2 (CK2) phosphorylation site Ser181, which can be decreased by treatment with CK2 inhibitor quinalizarin. Point mutation of the Ser181 phosphorylation site in MCF7/PGRMC1 cells impaired proliferation upon NET treatment. This study gives further insights into the mechanism of differential phosphorylation of the receptor and confirms our earlier hypothesis that phosphorylation of the CK2-binding site is essential for activation of PGRMC1. It further suggests an important role of PGRMC1 in the tumorigenesis and progression of breast cancer in progestin-based hormone replacement therapy.

## INTRODUCTION

Menopausal hormone therapy is administered for treatment of climacteric symptoms. The treatment can be carried out with the use of different estrogens, such as estradiol (E2), estriol (E3) or conjugated equine estrogens (CEE) [1]. In addition to estrogens, hormone therapy usually includes synthetic progestogens (progestins), which are added to prevent the development of endometrial hyperplasia and a consequent risk of endometrial cancer due to estrogen administration [2,3]. For sequential combined hormone therapy, which is applied mostly in peri- and early menopause, as well as for continuous combined hormone therapy preferably prescribed to postmenopausal women, special combined progestin-estrogen drugs were developed [1]. However, in important clinical studies, such as the Women's Health Initiative and the Million Women Study, combined estrogen-progestin therapy has been reported to be associated with a higher risk of breast cancer compared to estrogen-only therapy [4,5]. Various other studies also indicated a potential association between progestin treatment during menopause and increased breast cancer incidences [6–12].

Fournier et al. compared estrogen-only and different combined estrogen-progestin hormone therapies regarding breast cancer risk. In accordance with other studies, they reported that the risk of invasive breast cancer is significantly higher for hormone therapies using estrogen-progestin treatment than for estrogen-only therapies [12]. Further, they showed that the risk of breast cancer differs, depending on the type of progestin used. The orally administered progestins medrogestone, cyproterone acetate and norethisterone acetate were found to have the highest breast cancer risk. In contrast to combined estrogen-progestin therapy, no increased breast cancer risk has been reported for the combined therapy of estrogens and progesterone (P4) [12]. The impact of progestins on breast cells is broad and has not yet been fully elucidated. Progestins are referred to as structurally related to progesterone or testosterone. However, they differ from their molecule of origin and from each other in metabolism, pharmacokinetics, pharmacodynamics, potency and binding affinity to progesterone receptor (PR), causing different effects in breast cells [13,14]. Progestins are mainly designed as PR agonists to have anti-estrogenic actions in the endometrium [15]. However, they were also reported to display off-target effects by binding to androgen (AR), glucocorticoid (GR) and mineralocorticoid receptors (MR) with varying binding affinities [6,15–17]. Recent studies further indicate potential effects of progestins on Progesterone Receptor Membrane Component-1 (PGRMC1) [18–20]. PGRMC1 is a multifunctional protein and various cellular processes have been attributed to it, including heme-binding, binding and regulation of cytochrome P450 enzymes upon dimerization, P4 signaling and steroid response, vesicle trafficking and cell cycle regulation [21–34]. Moreover, PGRMC1 has been shown to be overexpressed in various cancer types, involved in cancer pathology and has been associated with increased tumor growth in progestin-based hormone therapy [19,35–39]. The diversity of PGRMC1 function might be regulated by a variety of posttranslational modifications, including phosphorylation, ubiquitination, acetylation and SUMOylation [40–42]. In a proteomics project, differential PGRMC1 phosphorylation in estrogen receptor-positive and -negative breast cancer has been observed, indicating that not only the expression level but also PGRMC1's phosphorylation may play a role in breast cancer [35]. Therefore, investigation of PGRMC1's regulation by posttranslational modifications, foremost PGRMC1 phosphorylation status, will further our knowledge about its biological functions and downstream signaling. According to the phosphosite database, the phosphorylation sites pS181, pY113, pS57 and pY180 are the most commonly observed sites [40,43].

In previous studies, we have shown that PGRMC1 is involved in the mode of action of progestins on breast cancer cells. We demonstrated that overexpression of PGRMC1 in MCF7 breast cancer cells (MCF7/PGRMC1) results in increased proliferation upon progestin treatment, as compared to empty vector control cells (MCF7/EVC) [36–38, 44–47].

As also observed in clinical studies, in our *in vitro* studies, various progestins exhibited different effects on proliferation of breast cancer cells. The progestins drospirenone, desogestrel, dydrogesterone, levonorgestrel, medroxyprogesterone acetate and norethisterone (NET) significantly increased the proliferation rate of MCF7/PGRMC1 cells, as compared to the control cells, whereas the progestins chlormadinone acetate and nomegestrel, as well as P4, did not increase proliferation at concentrations of 1 μM [36]. With significant impacts even at very low concentrations (0.01 μM and 0.1 μM), NET was shown to be the most potent progestin with regard to cell proliferation [36]. In *in vivo* studies, we could further demonstrate that a sequential combined treatment with E2 and NET significantly increased tumor growth of MCF7/PGRMC1 cells, compared to E2-only treatment, whereas MCF7/EVC cells did not respond to NET treatment [37]. Considering that PGRMC1 is expressed in breast tissue and overexpressed in breast cancer, further investigation of PGRMC1 activation and the resulting response of breast cancer cells is essential for the better understanding of the effects of progestins on breast cancer risk [37, 48–50].

To further study the biological activity of progestins associated with regulation of PGRMC1 activity, in the present study we investigated phosphorylation of PGRMC1 upon treatment with the progestin NET in PGRMC1-overexpressing MCF7- and T47D cells (T47D/PGRMC1). In addition, PGRMC1-overexpressing MCF7 cells exhibiting point mutations in relevant PGRMC1 phosphorylation sites were used to determine the significance of PGRMC1 phosphorylation for proliferation. For the first time, we show that PGRMC1 is phosphorylated at the Casein Kinase 2 (CK2) phosphorylation site Ser181 and is thus potentially activated by the progestin NET. Increasing concentrations of the CK2 inhibitor quinalizarin result in a significant decrease in PGRMC1 phosphorylation at Ser181, suggesting a role of this kinase in activation of the receptor. In addition, loss of the respective phosphorylation site significantly diminishes proliferation of PGRMC1-overexpressing MCF7 cells upon NET treatment. These results again indicate an important role of PGRMC1 in forwarding intracellular progestin signals in breast cancer cells and its contribution to the increased risk of breast cancer in progestin-based hormone therapy.

## RESULTS

### PGRMC1 overexpressing cells show increased proliferation upon NET treatment

**Figure 1:**
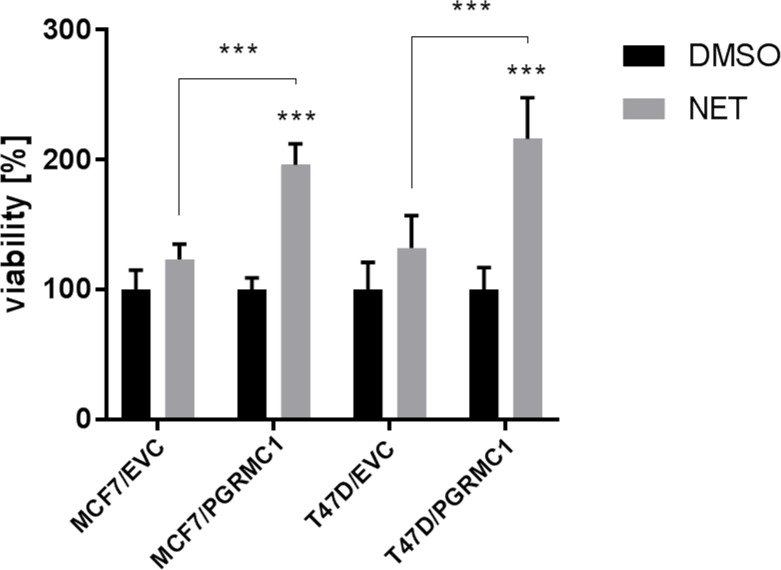
Proliferation of PGRMC1 overexpressing cells upon treatment with NET Treatment of MCF7/PGRMC1, MCF7/EVC, T47D/PGRMC1 and T47D/EVC cells with DMSO and NET for 72 h. Cell viability was normalized to corresponding DMSO control. Significantly increased proliferation of MCF7/PGRMC1 and T47D/PGRMC1 cells upon NET treatment compared to DMSO control and to NET-treated MCF7 and T47D vector control cells (***: p<0.005) (n = 3).

In previous studies, we reported increased proliferation of PGRMC1 overexpressing MCF7 cells upon treatment with the progestin NET compared to empty vector control cells. Now, we were able to confirm these results with PGRMC1 overexpressing T47D breast cancer cells (T47D/PGRMC1). Treatment of MCF7/PGRMC1 and T47D/PGRMC1 cells with NET for 72 h revealed significant increased proliferation as compared to cells treated with the DMSO control (p < 0.005) and 2-fold increased proliferation compared to NET-treated empty vector control cells (MCF7/EVC, T47D/EVC) (p < 0.005) (Figure 1).

**Figure 2:**
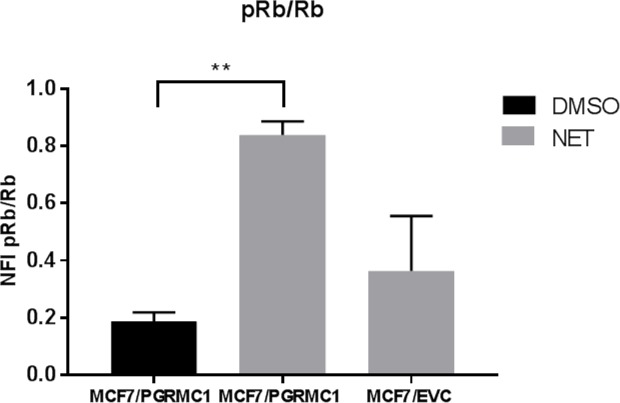
Abundance of Rb and pRb in MCF7 cells upon treatment with NET NFI signal ratios of p-Rb/Rb in MCF7/PGRMC1 cells treated with DMSO or NET and MCF7/EVC cells treated with NET for 72 h as measured by RPPA, (**: p<0.01).

Since the proliferation of PGRMC1 overexpressing cells is elevated upon treatment with NET, we investigated the effects on cell cycle proteins using Reverse Phase Protein Arrays (RPPA) technology. Treatment of MCF7/PGRMC1 cells resulted in a significantly increased phosphorylation of the tumor-suppressor protein and downstream cell cycle regulator Retinoblastoma protein (pRb), compared to DMSO-treated MCF7/PGRMC1 cells (p<0.005). In addition increased Rb phosphorylation between NET-treated MCF7/PGRMC1 and the vector control cells was observed-however, in this case, only with a strong tendency (p=0.077) (Figure 2).

### Detection of PGRMC1 phosphorylation sites

PGRMC1 phosphorylation was investigated in MCF7/PGRMC1 cells by mass spectrometry after immunoprecipitation of PGRMC1 from whole-cell lysates. Potential phosphorylation sites of PGRMC1 at Ser54, Ser57, Ser181, Thr178 and Tyr180 were identified (Supplementary Table 1). Further, using the scansite tool (http://scansite.mit.edu/), we searched for the protein kinases whereby the identified amino acids are phosphorylated and which domains they might bind to (Table 1) [51].

**Table 1:** PGRMC1 phosphorylation sites identified by mass spectrometry. PGRMC1 phosphorylation sites identified by mass spectrometry in MCF7/PGRMC1 cells. Localization probability indicates the probability of which the phosphorylation is the stated amino acid. Protein motifs as predicted from scansite website (http://scansite3.mit.edu/). (DNA PK: DNA-dependent serine/threonine protein kinase, SH2: Src homology 2, SHIP: SH2-containing inositol phosphatase)

Amino acid	Position	Sequence (localization probability)	Motif	Motif group	Stringency
S	S54	EGEEPTVY**S**(1)DEEEPKDESARK	DNA PK	DNA damage kinase group	low
S	S57	IVRGDQPAA**S**(0.863)GD**S**DDDEPPPLPR	Casein Kinase 2	Acidophilic serine/threonine kinase group	high
S	S181	GDQPAA**S**GD**S**(0.999)DDDEPPPLPR	Casein Kinase 2	Acidophilic serine/threonine kinase group	medium
T	T178	LLKEGEEP**T**(0.9)V**YS**DEEEPKDESAR	Casein Kinase 2	Acidophilic serine/threonine kinase group	low
Y	Y180	EGEEP**T**V**Y**(0.851**)S**DEEEPKDESAR	SHIP SH2	Src homology 2 group (SH2)	medium

### PGRMC1 is phosphorylated at Ser181 upon NET treatment

**Figure 3:**
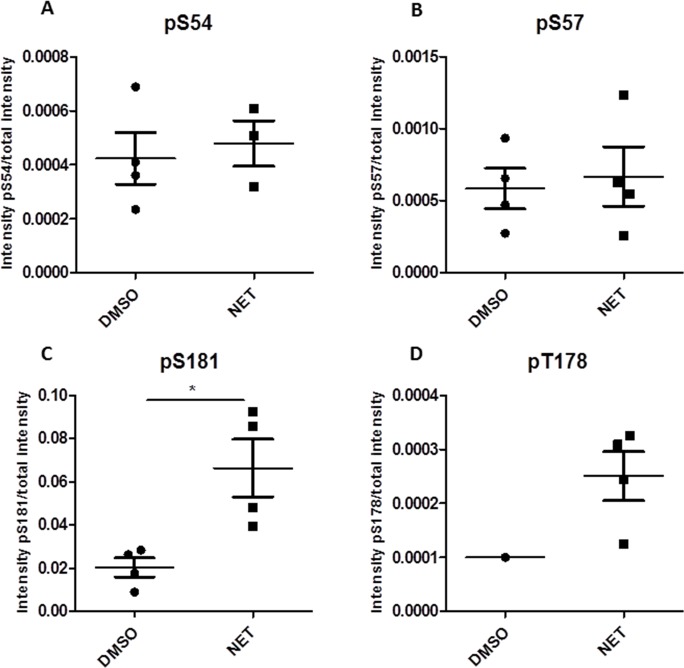
Intensity of phosphorylation in NET- and DMSO-treated MCF7/PGRMC1 cells after 72 h of treatment identified by mass spectrometry **(A)** S54 phosphorylation (DMSO: n = 4, NET: n = 4), **(B)** S57 phosphorylation (DMSO: n = 4, NET: n = 4), **(C)** S181 phosphorylation (DMSO: n = 4, NET: n = 4), (*: p<0.05), **(D)** T178 phosphorylation (DMSO: n = 1, NET: n = 4).

**Figure 4:**
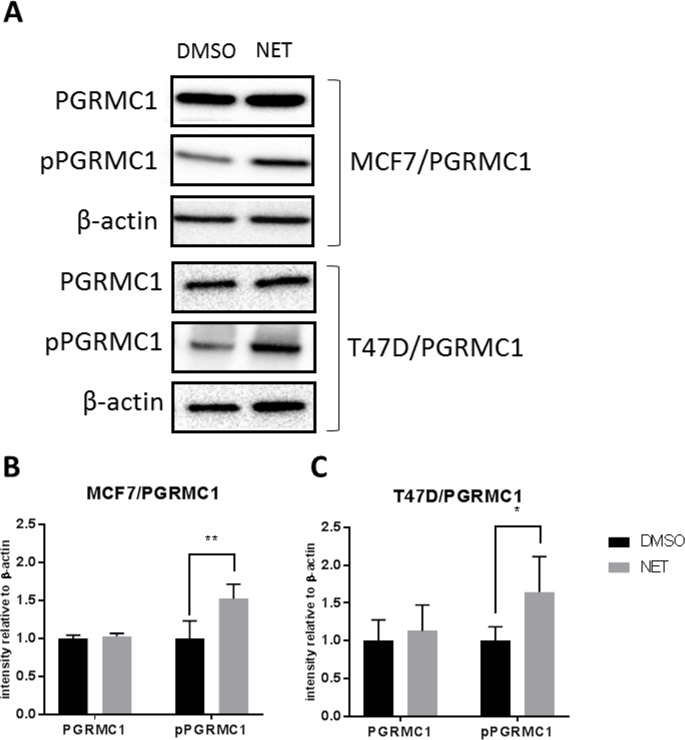
Phosphorylation of PGRMC1 at S181 upon NET treatment **(A)** Western blot analysis of PGRMC1 and pPGRMC1 in MCF7/PGRMC1 and T47D/PGRMC1 cells after 72 h of treatment with NET and DMSO. β-actin was used as loading control. **(B)** Densitometric analysis of Western blot results of MCF7/PGRMC1 cells treated with NET and DMSO (n = 3). Intensity was normalized to corresponding DMSO control. Significantly increased pPGRMC1 abundance in NET treated samples (**: p<0.01). **(C)** Densitometric analysis of Western blot results of T47D/PGRMC1 cells (n = 3). Intensity was normalized to corresponding DMSO control. Significantly increased pPGRMC1 abundance in NET treated samples (*: p<0.05).

Over recent years, we have investigated the impact of progestins on breast cancer, indicating a role of PGRMC1 in the signaling cascade after binding of progestins to hormone receptors [18–20, 35–38, 44–47, 52]. To investigate whether treatment with NET has an influence on PGRMC1 activation by induction of posttranslational modifications, the phosphorylation status of PGRMC1, immunopurified from DMSO- and NET-treated breast cancer cells, was determined by mass spectrometry and Western blot analysis. Comparison of NET- and DMSO-treated samples by mass spectrometry revealed no significant differences in phosphopeptide abundance for the phosphosites pS54, pS57 and pT178 (Figure 3A, 3B, 3D). A significantly higher relative signal intensity of the phosphopeptide EGEEPTVYpSDEEEPKDESARK (Ser181 phosphorylation site) for NET-treated samples, compared to DMSO-treated samples (Figure 3C, Supplementary Figure 1) (p = 0.0167) could be measured. Using an anti-phospho Ser181-PGRMC1 (anti-pPGRMC1) antibody, mass spectrometry results were validated by Western blot analysis in MCF7/PGRMC1- and T47D/PGRMC1 cells (Figure 4) and in MCF7 and T47D cells (Supplementary Figure 2). A significant higher PGRMC1 phosphorylation at Ser181 in NET-treated samples, as compared to DMSO-treated samples, could be identified (MCF7: p < 0.01, T47D: p < 0.05) (Figure 4). Expression levels of total PGRMC1 did not differ significantly in NET- and DMSO-treated MCF7 cells (Figure 4B) and T47D cells (Figure 4C).

### CK2 is involved in PGRMC1 phosphorylation at Ser181 upon NET treatment

**Figure 5:**
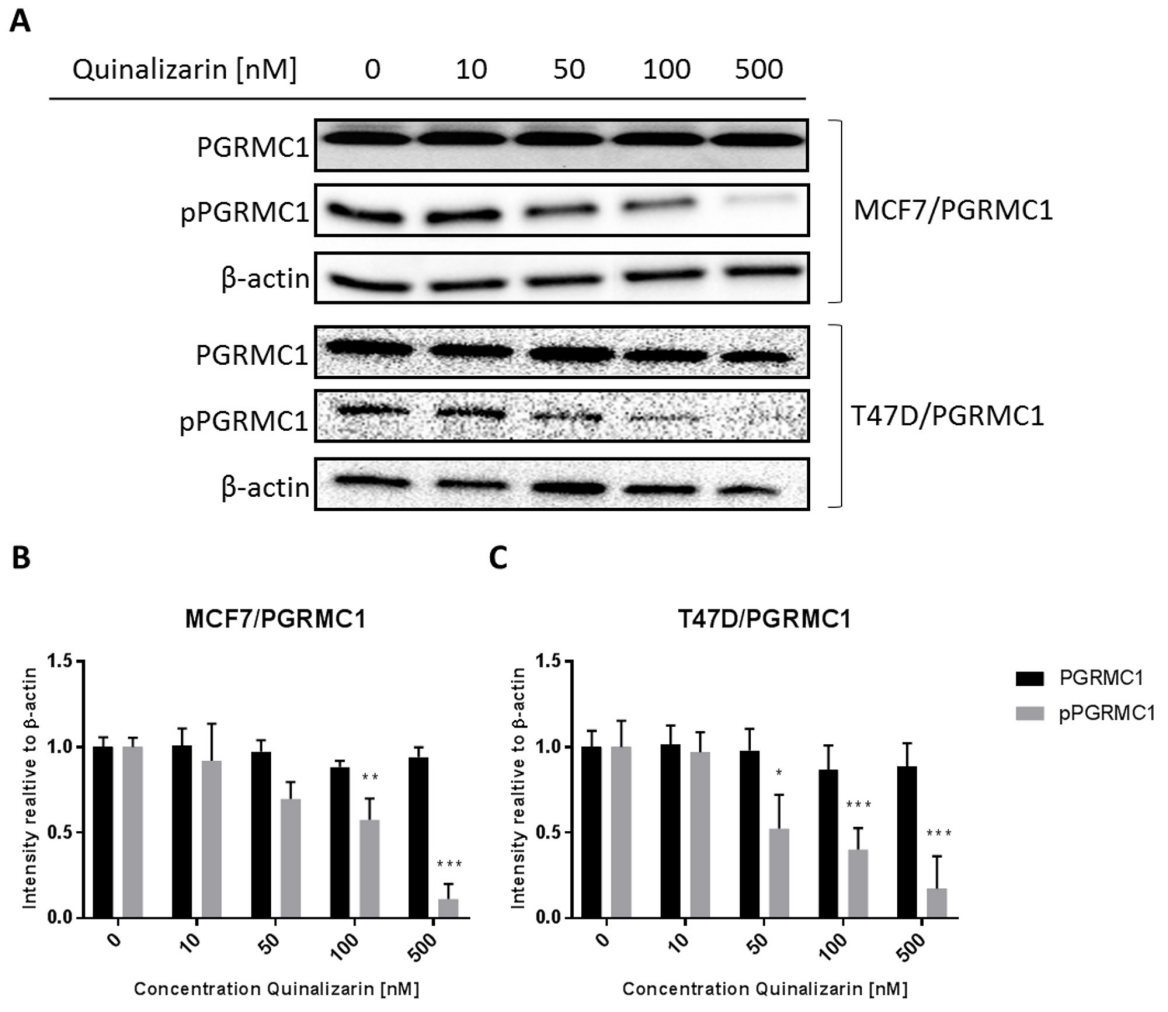
Phosphorylation of PGRMC1 at S181 upon treatment with NET and quinalizarin **(A)** Western blot analysis of PGRMC1 and pPGRMC1 after 24 h of treatment. MCF7/PGRMC1 and T47D/PGRMC1 cells were treated with NET and 0, 10, 50, 100 and 500 nM quinalizarin for 24 h. β-actin was used as loading control. **(B)** Densitometric analysis of Western blot results of MCF7/PGRMC1 cells (n = 3). Intensity was normalized to 0 nM quinalizarin. **(C)** Densitometric analysis of Western blot results of T47D/PGRMC1 cells. (n = 3). Intensity was normalized to 0 nM quinalizarin. (*: p<0.05, **: p<0.01, ***:<0.005)

Scansite analysis revealed that the Ser181 PGRMC1 phosphorylation site is likely to be phosphorylated by CK2 protein kinase (scansite score 0.498) [51]. Therefore, to examine the role of CK2 in PGRMC1 phosphorylation, we used the highly selective CK2 inhibitor quinalizarin (K_i_ = 520 nM) [53,54]. MCF7/PGRMC1- and T47D/PGRMC1 cells were treated with NET and increasing concentrations of quinalizarin. In the Western blot analysis the signals for pPGRMC1 decreased with increasing quinalizarin concentrations – reaching significance at 100 nM (p < 0.01) and 500 nM quinalizarin (p < 0.005) (Figure 5).

### PGRMC1 phosphorylation at Ser181 is essential for NET-induced proliferation of MCF7/PGRMC1 cells

**Figure 6:**
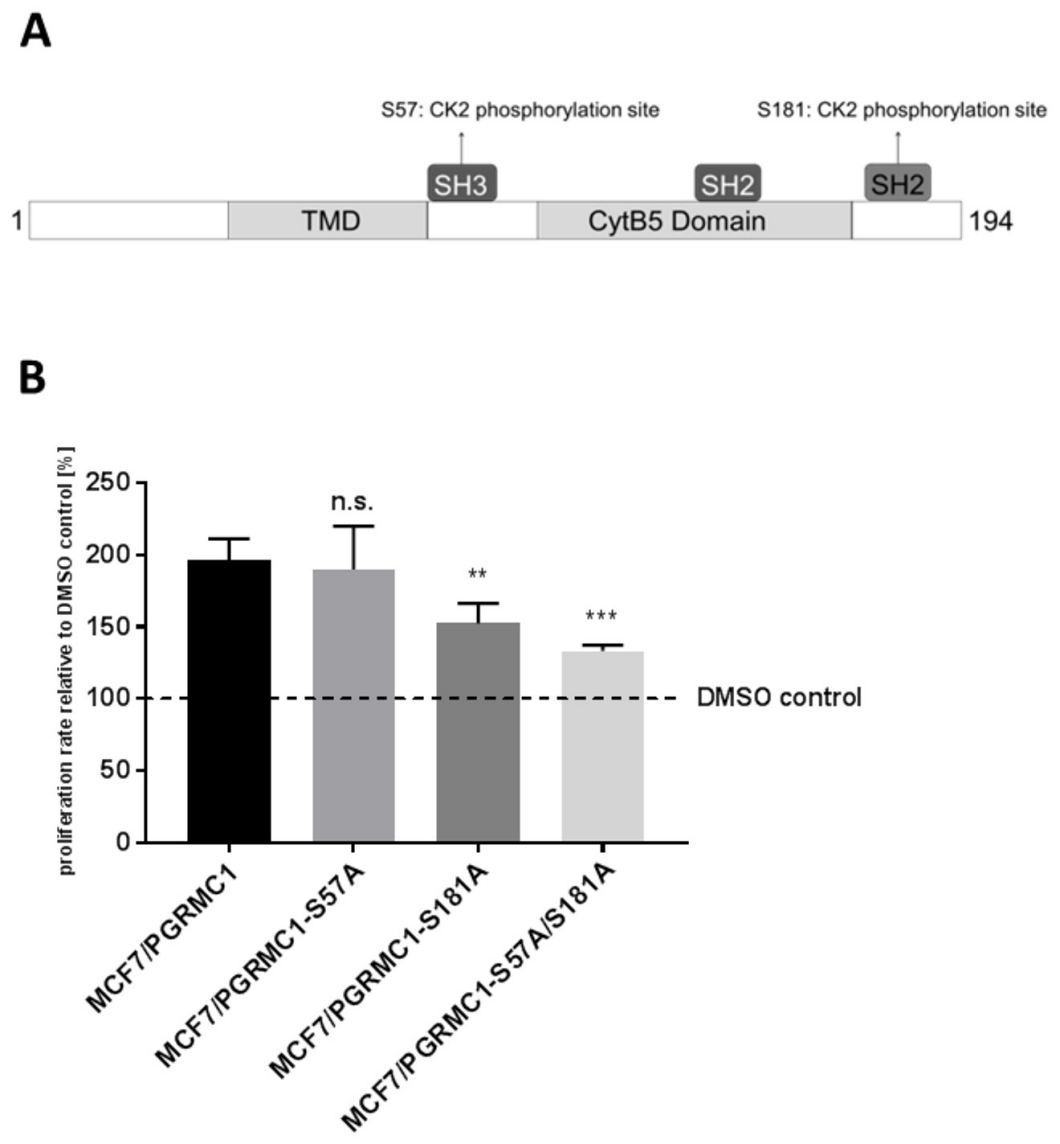
Point-mutation of S57 and S181 phosphorylation site **(A)** Schematic structure of PGRMC1 showing the point-mutated phosphorylation sites. **(B)** Proliferation of MCF7/PGRMC1, MCF7/PGRMC1-S57A, MCF7/PGRMC1-S181A and MCF7/PGRMC1-S57A/S181A cells after 72 h incubation with NET. Cell viability was normalized to corresponding DMSO control. (**: p<0.01, ***: p<0,005).

To further investigate the impact of PGRMC1 phosphorylation on cell proliferation, we stably transfected MCF7 cells with PGRMC1, exhibiting point mutations at the CK2 phosphorylation sites S57 (MCF7/PGRMC1-S57A) and S181 (MCF7/PGRMC1-S181A) and a double mutation at S57 and S181 (MCF7/PGRMC1-S57A/S181A) (Figure 6A). Cells were treated with NET for 72 h and cell viability was investigated by MTT assay (Figure 6B). Mutation at the S181 phosphorylation site, as well as double mutation at S181 and S57 phosphorylation sites resulted in significant lower proliferation rates after 72 h of stimulation (S181A: p < 0.01; S57A/S181A: p < 0.001), compared to MCF7 cells transfected with wild-type PGRMC1. No significant different proliferation rate could be detected for MCF7/PGRMC1-S57A cells, compared to MCF7/PGRMC1 cells.

## DISCUSSION

In various *in vitro* and *in vivo* studies, we investigated the effect of NET on PGRMC1-overexpressing MCF7 cells. These studies showed that, upon treatment with progestin, the proliferation rate and tumor volume are significantly increased compared to MCF7/EVC cells [36–38,44,47]. Here, we also report increased proliferation of T47D/PGRMC1 breast cancer cells upon treatment with NET compared to T47D/EVC cells, again supporting our previous results. These results indicate potential off target activities of NET on PGRMC1 causing increased proliferation. In T47D cells, NET was reported before to be bioconverted into the a-ring reduced metabolites 3α,5α-norethisterone and 5α-norethisterone potentially by 5 alpha-steroid reductase and aldo-keto reductases. While NET is a PR-agonist, 3α,5α-norethisterone and 5α-norethisterone are ER-agonists [55,56]. Abundance of 5 alpha-steroid reductase was reported before for MCF7 cells, as well [57]. Therefore, it is also conceivable that instead of NET its metabolites might induce the described effects on PGRMC1 overexpressing cells. Furthermore, on the molecular level, an elevated level of phosphorylated Rb was detected in MCF7/PGRMC1 cells treated with NET compared to MCF7/EVC and DMSO treated cells, indicating an influence of PGRMC1 activation on cell cycle regulation. The tumor-suppressor protein Rb is known to regulate cell proliferation by controlling progression through the G1/S phase checkpoint of the cell cycle. Phosphorylation by cyclin-dependent kinases inhibits Rb and allows cell cycle progression [58,59]. A role of PGRMC1 in cell cycle progression has previously been reported [31,33,60]. Ahmed et al. demonstrated that inhibition of PGRMC1 by AG-205 induces G1 cell cycle arrest, indicating a role of the receptor in this phase of the cell cycle [31]. Our results are in accordance with these findings and suggest that the reported correlation between PGRMC1 and cell cycle progression is caused by phosphorylation of Rb upon PGRMC1 activation.

PGRMC1 is a multifunctional protein and its diverse functions were suggested to be regulated by a variety of posttranslational modifications, foremost by its phosphorylation [40]. Further, as recently reviewed by Cahill et al., PGRMC1 phosphorylation might play a crucial role not only in terms of its function, but also in its interaction and subcellular localization [21,24,26,40,41]. In the present study, we investigated PGRMC1 phosphorylation at S54, S57, S181, T178 and Y180. In the past, PGRMC1 has been shown to exhibit various phosphorylation sites. According to the phosphosite database, pS181, pY113, pS57 and pY180 are the most commonly observed sites [40,43]. Phosphorylation site Y180 is a potential SH2-target sequence, requiring tyrosine phosphorylation for SH2 protein domains to bind and thereby inducing conformational changes of the receptor. However, phosphorylation of S181 and T178 by CK2 is predicted to sterically inhibit phosphorylation of Y180 and to attenuate protein interaction, which might explain the low signals for the pY180 phosphosite in the present study [40,41]. Likewise, binding of SH3 domains to SH3 target sequence P63 might be affected by phosphorylation of S57 by CK2 [21,40]. Although phosphorylation of Y113 is reported to be among the most common PGRMC1 phosphorylation sites, in the present study phosphorylation of Y113 could not be detected by mass spectrometry [43]. Phosphorylation of Y113 might be responsible for the membrane trafficking function of PGRMC1, but at the same time prevent heme-binding due to steric interference, suggesting a reciprocal regulation [24,40]. It has recently been shown that upon heme-binding, PGRMC1 forms dimers mediated by dephosphorylated Y113 and that receptor dimerization is required for activation of CYP450 enzymes [24].

To gain deeper insights into the mechanism of action of progestins on PGRMC1, we aimed to further investigate PGRMC1 signaling upon NET treatment in the present study. For the first time, we could show that PGRMC1 is phosphorylated at S181 and thus probably activated by the progestin NET. As known for other steroid receptors, in the presence of NET or its metabolites PGRMC1 might potentially undergo conformational changes, which exposes the phosphorylation site or induces dissociation from chaperones, enhancing phosphorylation of the receptor by kinases [61,62]. Serine 181 is the most commonly observed phosphosite of PGRMC1 and is predicted to be phosphorylated by CK2, a constitutively active kinase responsible for phosphorylation of a large proportion of proteins [43,63]. Cahill et al. suggested that the Y180 and the S181 phosphorylation sites are accessible for protein interactions, since they are located in unstructured regions of the protein, which were unstable under NMR conditions [24,40,41]. Thus, increased phosphorylation of PGRMC1 at S181 upon NET treatment might lead to augmented recruitment of enzymes or protein-protein interactions, resulting in altered signal transduction [40].

Besides characterization of PGRMC1 phosphorylation status, also identification of kinases and phosphatases responsible for phosphorylation and dephosphorylation is important to better understand PGRMC1 signaling. Simultaneous treatment of MCF7/PGRMC1- and T47D/PGRMC1 cells with NET and quinalizarin, a cell-permeable highly specific CK2 inhibitor, resulted in a decrease of S181 phosphorylation [53,54]. This result confirms the assumption that CK2 is responsible for, or involved in, phosphorylation of PGRMC1 at S181. Our results are supported by a recently published quantitative phosphoproteome analysis using quinalizarin to detect CK2 target proteins. In this study, for the first time PGRMC1 was shown to be a target of CK2. They showed immunoprecipitated PGRMC1 to be phosphorylated in the presence of CK2, while in the absence of CK2 no PGRMC1 phosphorylation could be detected. Treatment with 1 μM quinalizarin revealed decreased phosphorylation [64].

In previous studies, we showed that PGRMC1 mutation at S57 and S181 results in a modified function of PGRMC1 in MCF7 cells. Upon H_2_O_2_ exposure, susceptibility to cell death of stably PGRMC1-overexpressing MCF7 cell lines carrying point mutations at S57 and S181 was altered, as well as phosphorylation of Akt [35]. Here we could show that mutation of S181 and double mutation of S57 and S181 diminished susceptibility of MCF7/PGRMC1 cells to NET treatment. The proliferation rate of these cells upon NET treatment was comparable to empty vector control cells, whereas sole mutation of S57 did not diminish cell proliferation. This indicates that PGRMC1 phosphorylation at S181 phosphorylation site is crucial for NET-induced downstream signaling of PGRMC1. Various attempts to explain this observed effect are conceivable. As discussed above, phosphorylation of S181 might induce protein-protein interactions between the receptor and downstream signaling proteins. Mutation of the amino acid, and thus deletion of the respective phosphorylation site, might therefore prevent downstream signaling. As discussed elsewhere, another approach could be that deletion of S57 and S181 phosphorylation sites enables phosphorylation of P63 and Y180, which is otherwise sterically inhibited by pS57 and pS181 [21,40,41]. Phosphorylation of P63 and Y180 could then in turn lead to recruitment of SH3- and SH2-target proteins, resulting in altered downstream signaling. Further, a lack of S57 and S181 phosphorylation and thus a gain of P63 and Y180 phosphorylation might induce conformational changes of PGRMC1, causing altered biological functions of the receptor.

In the present study, we were able to gain further insights into the mechanisms of receptor activation upon treatment with the progestin NET which requires/involves differential phosphorylation of PGRMC1. It again demonstrates a role of PGRMC1 in transducing progestin signals in breast cancer cells. The characterization of the PGRMC1 phosphorylation state and associated function requires further investigation and is important for a better understanding of the involvement of PGRMC1 in increased breast cancer risk in progestin-based hormone therapy.

## MATERIALS AND METHODS

### Cell culture

MCF7 and T47D cells were obtained from ATCC (Manassas, Virginia) and authenticated by Microsynth AG (Balgach, Switzerland) on October 7, 2016. Profiling of human cell lines was done using highly-polymorphic short tandem repeat loci (STRs). STR loci were amplified using the PowerPlex® 16 HS System (Promega, Madison, Wisconsin). Fragment analysis was done on an ABI3730xl (Thermo Fisher Scientific, Waltham, Massachusetts) and the resulting data were analyzed using GeneMarker HID software (Softgenetics, State College, Pennsylvania).

Cells were stably transfected with expression plasmid pcDNA3.1 containing hemagglutinin (HA)-tagged PGRMC1 wild-type or HA-tagged PGRMC1 mutants S57A, S181A and S57A/S181A, as described elsewhere [35]. MCF7 and T47D cells were maintained in RPMI 1640 medium (Thermo Fisher Scientific, Waltham, Massachusetts), supplemented with 10% fetal bovine serum (Thermo Fisher Scientific, Waltham, Massachusetts), 100 units/ml penicillin/streptomycin (Thermo Fisher Scientific, Waltham, Massachusetts) and 25 mM HEPES (Thermo Fisher Scientific, Waltham, Massachusetts) in a humidified incubator at 37°C with 5% CO_2_.

### Treatment of cells

For NET treatment, cells were seeded in complete medium. After 48 h, the medium was changed to a steroid-free medium, using a phenol-red free RPMI 1640 medium (Thermo Fisher Scientific, Waltham, Massachusetts), 10% charcoal stripped fetal bovine serum (Thermo Fisher Scientific, Waltham, Massachusetts), 100 units/ml penicillin/streptomycin and 25 mM HEPES and incubated for another 48 h. Treatment was performed with 1 μM NET (Sigma-Aldrich, St. Louis, Missouri) or 0.01% DMSO (Sigma-Aldrich, St. Louis, Missouri) as a control, for a defined time period in a steroid-free medium. To investigate signaling of PGRMC1 phosphorylation, simultaneous to treatment with NET, cells were treated with the CK2 inhibitor quinalizarin (Sigma-Aldrich, St. Louis, Missouri) in concentrations of 10, 50, 100, 500 nM and the respective DMSO volume as a control for 24 h. For cell harvest after treatment, cells were washed with ice-cold PBS (Thermo Fisher Scientific, Waltham, Massachusetts) twice, harvested using cell lifter (Corning, Tewksbury, Massachusetts) and transferred into an Eppendorf tube.

### (Co-)Immunoprecipitation

For immunoprecipitation, MCF7/PGRMC1 cells were lysed using a mild lysis buffer (20 mM TRIS (Sigma-Aldrich, St. Louis, Missouri), 137 mM NaCl (Sigma-Aldrich, St. Louis, Missouri), 1% Nonidet P 40 Substitute (NP-40) (Sigma-Aldrich, St. Louis, Missouri), 2 mM EDTA (Sigma-Aldrich, St. Louis, Missouri) containing protease- and phosphatase inhibitors (Roche, Basel, Switzerland). Immunoprecipitation was performed using Pierce™ HA-Tag IP/Co-IP Kit (Thermo Fisher Scientific, Waltham, Massachusetts) according to the manufacturer's instructions.

### Mass spectrometry

PGRMC1 was immunoprecipitated from four individual replicates of DMSO and four individual replicates of NET-treated MCF7/PGRMC1 cells. The resulting protein preparations were rapidly separated in a 4–12% polyacrylamide gel (about 4 mm running distance), silver stained and processed as previously described [65]. Briefly, samples were destained, reduced with dithiothreitol, alkylated with iodoacetamide, digested with trypsin (Serva, Heidelberg, Germany) and peptides extracted from the gel and finally resuspended in 0.1% trifluoroacetic acid. Subsequently, the samples were analyzed on a liquid chromatography coupled electrospray ionization Orbitrap mass spectrometer. An Ultimate 3000 Rapid Separation Liquid Chromatography System (RSLC, Thermo Fisher, Dreieich, Germany) was used for peptide separation: peptides were initially pre-concentrated on a trap column (Acclaim PepMap100, 3 μm C18 particle size, 100 Å pore size, 75 μm inner diameter, 2 cm length, Thermo Scientific, Dreieich, Germany) at a flow rate of 6 μl/min for 10 min, using 0.1% TFA as mobile phase and thereafter separated on an analytical column (Acclaim PepMapRSLC, 2 μm C18 particle size, 100 Å pore size, 75 μm inner diameter, 25 cm length, Thermo Scientific, Dreieich, Germany) at a flow rate of 300 nl/min at 60°C, using a 2 h gradient from 4 to 40% solvent B (0.1% (v/v) formic acid, 84% (v/v) acetonitrile in water) in solvent A (0.1% (v/v) formic acid in water). The liquid chromatography system was online coupled to an Orbitrap Elite mass spectrometer (Thermo Scientific, Dreieich, Germany) via a nano electrospray ionization source and peptides injected by distal coated Silica Tip emitters (New Objective) using a spray voltage of 1.45 kV. The mass spectrometer was operated in positive, data-dependent mode with capillary temperature set to 225°C. Firstly, full scans (350-1700 m/z, resolution 60000) were recorded in the Orbitrap analyzer of the instrument with a maximal ion time of 200 ms and the target value for automatic gain control set to 1000000. In the linear ion trap part of the instrument, subsequently up to 20 double- and triple-charged precursors with a minimal signal of 500 were isolated (isolation window 2 m/z), fragmented by collision-induced dissociation (CID) and analyzed with a maximal ion time of 50 ms and the target value for automatic gain control set to 3000 (available mass range 50–2000 m/z, resolution 5400). Already analyzed precursors were excluded from further isolation and fragmentation for 45 seconds.

For data analysis, the MaxQuant environment (version 1.5.3.8, Max Planck Institute of Biochemistry, Planegg, Germany) was used, with standard parameters unless otherwise stated. Spectra were searched against 20187 Swiss-Prot entries from the Homo sapiens proteome (UP000005640, downloaded on November 18, 2015 from UniProt KB). Label-free quantification was enabled, as well as the ‘match between runs’ option. Tryptic cleavage specificity was chosen, as well as carbamidomethyl at cysteines as fixed and methionine oxidation, phosphorylation (threonine, serine and tyrosine), acetylation at protein N-termini and ubiquitination at lysine (GlyGly, +114.0429) as variable modifications. Mass tolerances were 20 ppm (first search) and 4.5 ppm (second search after recalibration) for precursor masses, and 0.5 Da for fragment masses. Phosphorylation sites were reported showing the highest probability calculated form and MS/MS spectrum peak matches. Peptides and proteins were accepted at a false discovery rate of 1%. For relative quantification of phosphorylated peptides, peptide intensities were normalized to progesterone receptor amounts by dividing them by the total progesterone receptor intensity.

### Western blot analysis

Cells were lysed in RIPA lysis buffer (50 mM TRIS, 150 mM NaCl, 1% NP-40, 0.5% sodium deoxycholat (Merck KGaA, Darmstadt, Germany), 0.1% SDS (Sigma-Aldrich, St. Louis, Missouri) containing protease and phosphatase inhibitors. Protein concentration was determined using Pierce™ BCA Protein Assay Kit (Thermo Fisher Scientific, Waltham, Massachusetts). 25 μg of total protein was loaded onto Mini-PROTEAN^®^ TGX™ precast gels (Bio-Rad Laboratories, Inc., Hercules, California). Protein samples were separated by polyacrylamide gel electrophoresis and transferred to Immun-Blot^®^ PVDF Membranes (Bio-Rad Laboratories, Inc., Hercules, California). Membranes were blocked with 5% skim milk powder (Sigma-Aldrich, St. Louis, Missouri) in TRIS-buffered saline containing 0.1% Tween20 (Sigma-Aldrich, St. Louis, Missouri) (TBS-T) for 60 min at room temperature and incubated with respective primary antibodies overnight at 4°C. After washing membranes with TBS-T, secondary antibodies were applied in 1% skim milk powder/TBS-T and incubated at room temperature for 1 h. Membranes were again washed with TBS-T and ECL reagent (GE Healthcare, Little Chalfont, UK) was applied prior to chemiluminescent imaging, using ChemiDoc^TM^ MP-System (Bio-Rad Laboratories, Inc., Hercules, California). Primary antibody against PGRMC1 (Cell Signaling Technology, Cambridge, UK) and β-actin (Santa Cruz, Dallas, Texas) and respective HRP-conjugated secondary goat-anti-rabbit and goat-anti-mouse antibodies (Santa Cruz, Dallas, Texas) were used according to the manufacturer's recommendations. Primary antibody against pPGRMC1 was manufactured by ProteoSys AG (Mainz, Germany) and validated as previously described [39].

### MTT assay

Cells (5 × 10^4^ cells per well) were first cultured in 96-well plates in complete medium for 48 h, then treated with steroid-free medium for 48 h, followed by NET treatment for 72 h, as described above. For cell viability assay after treatment with NET, the medium was aspirated, cells were washed with PBS. 100 μl of steroid-free medium, supplemented with 0.25 mg/ml Thiazolyl Blue Tetrazolium Bromide (MTT) (Sigma-Aldrich, St. Louis, Missouri), was added per well. After 3 h of incubation at 37°C, MTT solution was aspirated and 100 μl DMSO (Sigma-Aldrich, St. Louis, Missouri) was added. Following 1 h of incubation at 37°C, absorbance was measured at 540 nm, using a microplate reader (anthos Reader HT2, Anthos Mikrosysteme GmbH, Krefeld, Germany).

### Reverse phase protein arrays

Reverse Phase Protein Arrays (RPPA) using Zeptosens technology (Bayer AG, Leverkusen, Germany) were used for analysis of signaling protein expression and activity profiling as described earlier [66–69].

For the analysis, flash frozen cell pellets were lysed by incubation with 100 μl cell lysis buffer CLB1 (Bayer AG, Leverkusen, Germany) for 30 min at room temperature. Total protein concentrations of the lysate supernatants were determined by Bradford Assay (Coomassie Plus, Thermo Scientific, Waltham, Massachusetts). Cell lysate samples were adjusted to uniform protein concentration in CLB1, diluted 10-fold in RPPA spotting buffer CSBL1 (Bayer AG, Leverkusen, Germany) and subsequently printed as series of four dilutions (starting concentration at 0.3 μg/μl plus 1.6-fold dilutions) and in two replicates each. All samples were printed as replicate microarrays on to Zeptosens hydrophobic chips (Bayer AG, Leverkusen, Germany) using a NanoPlotter 2 (GeSim, Grosserkmannsdorf, Germany) applying single droplet depositions (0.4 nL volume per spot). After printing, the microarrays were blocked with 3% w/v albumin, washed thoroughly with double distilled H_2_O, dried in a stream of nitrogen and stored in the dark at 4°C until further use. Protein expression and activity levels were measured using a direct two-step sequential immunoassay and sensitive, quantitative fluorescence read-out. A single array was probed for each protein. Highly specific and upfront validated primary antibodies were incubated at the respective dilution in Zeptosens assay buffer overnight (15 h) at room temperature. Arrays were washed once in assay buffer and incubated for 45 min with Alexa647-labeled anti-species secondary antibody (Invitrogen, Paisley, UK). Arrays were then washed as before and imaged, using a ZeptoREADER instrument (Bayer AG, Leverkusen, Germany) in the red laser channel. Typically, six fluorescence images were recorded for each array at exposure times of between 0.5 and 16 seconds. Negative control assays incubated in the absence of primary antibody (blank assays) were performed to measure the non-specific signal contributions of the secondary antibody. In addition, one chip out of the print series was stained to measure the relative amount of immobilized protein per spot (protein stain assay). The following primary antibodies (provider and reagent number, dilution) were used: Rb (Cell Signaling Technology, Cambridge, UK, CST 9309, 1:200), Rb-P-Ser807/Ser811 (Cell Signaling Technology, Cambridge, UK, CST 8516, 1:100). RPPA assay images were analyzed, using ZeptoVIEW Pro 3.1 array analysis software (Bayer AG). Sample signals were quantified as protein–normalized, blank-corrected mean fluorescence intensities (NFI) of the single spots applying linear fits and interpolating to the mean of the four printed sample dilutions (eight spots per sample).

### Statistical analysis

Densitometric analysis of PGRMC1 and pPGRMC1 immunoblots was performed on scanned immunoblot images, using the ImageJ gel analysis tool [70]. The gel analysis tool was used to obtain the absolute intensity for each band and corresponding actin band. The relative intensity of each band representing PGRMC1 and pPGRMC1 was calculated by normalizing the absolute intensity of the band to the corresponding control band. All experiments were repeated a minimum of three times. Differences between treated and untreated groups were determined by Student's t-test using Graph Pad Prism (GraphPad Software, Inc.).

## SUPPLEMENTARY MATERIALS FIGURES AND TABLE


