# Supplementary material for: Progesterone receptor membrane component 1 is phosphorylated upon progestin treatment in breast cancer cells
Source: Oncotarget. 2017 Aug 2;8(42):72480–93. doi: 10.18632/oncotarget.19819 (PMC5641147; doi:10.18632/oncotarget.19819)
Supplement: Supplementary file 1 [file oncotarget-08-72480-s001.pdf]

# Progesterone receptor membrane component 1 is phosphorylated upon progestin treatment in breast cancer cells

## SUPPLEMENTARY MATERIALS

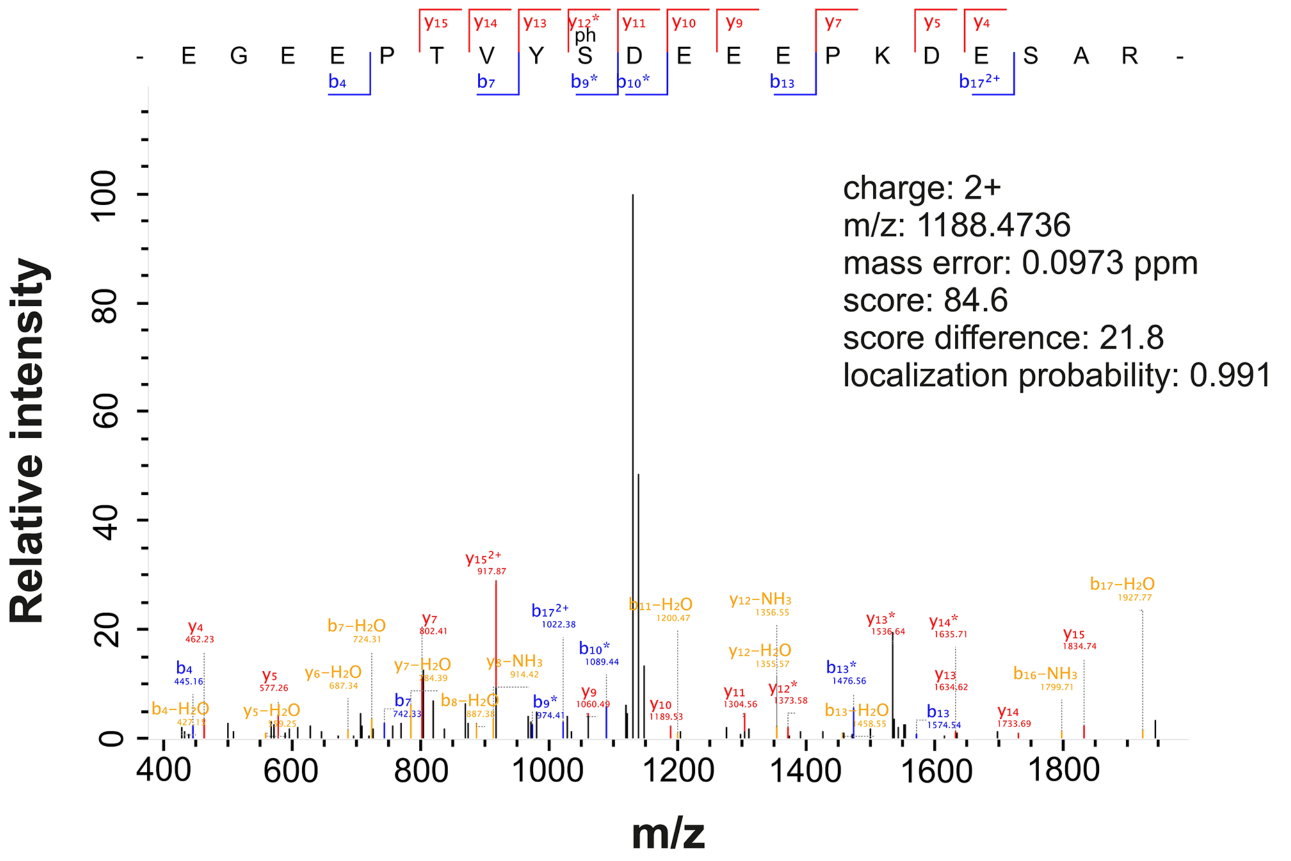


**Supplementary Figure 1: Annotated MS/MS spectrum of a 1188.** 4736 m/z two-fold charged precursor, fragmented by CID which can be attributed to the sequence EGEEPTVYSDEEEPKDESARK including one phosphorylation. A phosphorylation at S181 shows highest score resulting in a localisation probability of 99%.

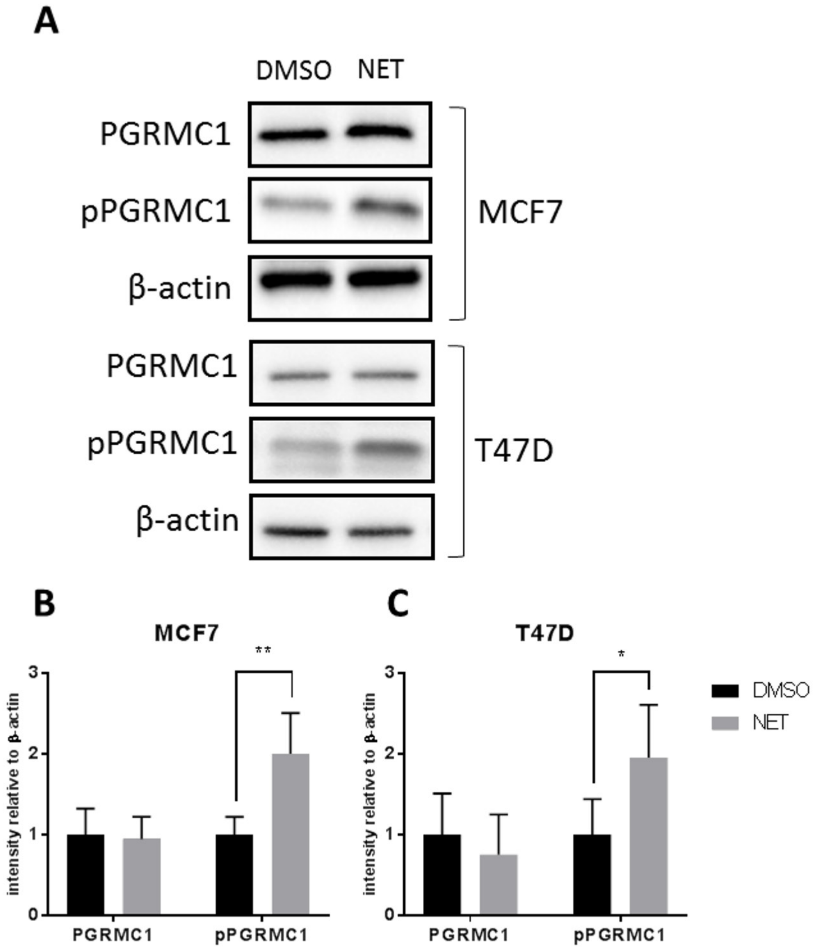


**Supplementary Figure 2: Phosphorylation of PGRMC1 at S181 upon NET treatment in MCF7 and T47D cells. (A)** Western blot analysis of PGRMC1 and pPGRMC1 in MCF7 and T47D cells after 72 h of treatment with NET. β-actin was used as loading control. **(B)** Densitometric analysis of Western blot results of MCF7 cells (n = 3). Intensity was normalized to corresponding DMSO control. Significantly increased pPGRMC1 abundance in NET treated samples (**: $p<0.01$). **(C)** Densitometric analysis of Western blot results of T47D/PGRMC1 cells (n = 3). Intensity was normalized to corresponding DMSO control. Significantly increased pPGRMC1 abundance in NET treated samples (*: $p<0.05$).

**Supplementary Table 1: Phosphopeptides were detected using liquid chromatography coupled mass spectrometry. Sites were localized according to the fragment spectra matching pattern. Scores and probabilities are given for peptide and phospho site identification and localisation**

| Phospho (STY) Probabilities | Mass error [ppm] | Score diff | Posterior error probability | Score | Score for localization | Delta score | Localization probability |
|---|---|---|---|---|---|---|---|
| EGEEPTVYS(1) DEEEPKDESARK | -0.569 | 38.8 | 2.75E-36 | 136.2 | 121.9 | 96.6 | 0.9998 |
| IVRGDQPAAS(0.863) GDS(0.137) DDDEPPPLPR | 0.397 | 8.0 | 2.91E-19 | 111.4 | 74.9 | 77.6 | 0.8626 |
| GDQPAAS(0.001) GDS(0.999) DDDEPPPLPR | -0.085 | 32.0 | 2.76E-27 | 120.2 | 114.3 | 75.7 | 0.9994 |
| LLKEGEEPT(0.9) VY(0.042)S(0.058) DEEEPKDESAR | 1.112 | 11.9 | 3.85E-09 | 93.2 | 54.3 | 65.1 | 0.9001 |
| EGEEPT(0.031) VY(0.851)S(0.117) DEEEPKDES(0.002) AR | 0.176 | 8.6 | 0.0046791 | 50.1 | 50.1 | 28.1 | 0.8511 |
